# The PRINTO provisional definition of remission in juvenile dermatomyositis

**DOI:** 10.1186/1546-0096-9-S1-P194

**Published:** 2011-09-14

**Authors:** D Lazarevic, A Pistorio, P Miettunen, A Ravelli, C Malattia, C Pilkington, N Wulffraat, S Garay, M Hofer, P Quartier, P Dolezalova, I Calvo Penades, V Ferriani, G Ganser, O Kasapcopur, JA Melo-Gomes, M Wierzbowska, A Martini, N Ruperto

**Affiliations:** 1 IRCCS G. Gaslini, Pediatria II, PRINTO, Italy

## Objective

To define a definition of remission in juvenile dermatomyositis (JDM) that is stringent but achievable and could be applied uniformly as an outcome measure in clinical trials.

## Methods

275 patients in active phase of JDM <18 years, median disease duration 7.7 months, were evaluated at baseline and 24 months. Out of 275 patients, all patients (39/275) who were off treatment at 24 months were defined as being in clinical remission and were included as the “gold standard” group. A random sample of patients (n=78) who were in an active phase of JDM at baseline and on medications at 24 months, were selected as the “comparison group”. Literature was reviewed for definitions of remission and in addition other definitions were tested which included PRINTO core set variables, muscle enzymes, and other JDM activity measures. Accuracy measurements were calculated: sensitivity, specificity, Youden index, Cohen’s kappa (≥0.8 almost perfect).

## Results

Four best single variables with kappa≥0.8 were Manual Muscle Testing (MMT)≥78 (sensitivity: 0.92, specificity 0.97, kappa 0.9), followed by Physician Global Assessment of Muscle Activity (PhyMAVAS) and Physician Global Visual Analogue Scale (PhyGloVAS)≤0.2 and Childhood Myositis Assessment Scale (CMAS)≥48. The most accurate combination of variables with kappa≥0.8 included 4 variables with 3 out of 4 required to be present: creatine phosphokinase ≤150, CMAS≥48, MMT≥78, PhyGloVAS≤0.2 (sensitivity 0.94, specificity 1.00, kappa 0.96).

## Conclusion

The combination definition performed the best overall in defining remission in JDM and could be used for the evaluation of global response to therapy in future clinical trials.

